# Inclusion of a dual signal sequence enhances the immunogenicity of a novel viral vectored vaccine against the capsular group B meningococcus

**DOI:** 10.1186/s13578-022-00809-3

**Published:** 2022-06-11

**Authors:** Dylan Sheerin, Christina Dold, Laura Silva-Reyes, Aline Linder, Andrew J. Pollard, Christine S. Rollier

**Affiliations:** 1 Oxford Vaccine Group, Department of Paediatrics, University of Oxford, and the NIHR Oxford Biomedical Research Centre, Centre for Clinical Vaccinology and Tropical Medicine, Churchill Hospital, Oxford, UK; 2 Infectious Diseases and Immune Defence Division, Institute of Medical Research (WEHI), The Walter & Eliza Hall, Parkville, VIC 3052 Australia; 3 Present Address: Faculty of Health and Medical Sciences, University of Surrey, Guildford, UK

**Keywords:** Meningococcal disease, Viral vector vaccines, Signal sequence, Transgene, Expression kinetics

## Abstract

**Background:**

Disease caused by the capsular group B meningococcus (MenB) is the leading cause of infectious death in UK infants. A novel adenovirus-based vaccine encoding the MenB factor H binding protein (fHbp) with an N-terminal dual signal sequence induces high titres of protective antibody after a single dose in mice. A panel of N-terminal signal sequence variants were created to assess the contribution of components of this sequence to transgene expression kinetics of the encoded antigen from mammalian cells and the resultant effect on immunogenicity of fHbp.

**Results:**

The full-length signal sequence (FL SS) resulted in superior early antigen expression compared with the panel of variants, as measured by flow cytometry and confocal imaging, and supported higher bactericidal antibody levels against the expressed antigen in mouse sera < 6 weeks post-immunisation than the licensed four component MenB vaccine. The FL SS also significantly increased antigen-specific T cell responses against other adenovirus-encoded bacterial antigens in mice.

**Conclusions:**

These findings demonstrate that the FL SS enhances immunogenicity of the encoded antigen, supporting its inclusion in other viral vectored bacterial antigen transgenes.

**Supplementary Information:**

The online version contains supplementary material available at 10.1186/s13578-022-00809-3.

## Introduction

The MenB capsular polysaccharide is poorly immunogenic [1], and therefore efforts to develop a capsular group B meningococcal (MenB) vaccine have focused primarily on the identification of immunogenic surface-exposed proteins. The vaccines currently licensed to prevent MenB infection—the four component MenB vaccine, 4CMenB [2], and the bivalent fHbp vaccine, MenB-fHbp [3]—require multiple doses to achieve clinical protection [4], rendering a 4CMenB or MenB-fHbp immunisation campaign not cost-effective for the adolescent population in the UK [5]. There is also concern about the long-term persistence of serum bactericidal antibody (SBA) titres, and thus the duration of protection; waning protective responses in the years following the primary vaccination series of both 4CMenB and MenB-fHbp and the robust immune responses elicited after a 48 month booster dose suggest a need to consider whether sufficient protection is conferred by existing MenB immunisation schedules [6–8]. Therefore, alternative MenB vaccines that are well tolerated and capable of inducing protective immune responses without the need for multiple doses, and which confer persistent protection, are needed to simplify MenB immunisation schedules and provide better duration of protection, which in turn might make MenB immunisation campaigns more likely to be cost-effective.

The surface-exposed meningococcal lipoprotein, fHbp, sequesters host factor H during the invasive stage of infection and therefore plays a crucial role in meningococcal serum resistance [9]. All invasive *Neisseria meningitidis* capsular group B strains encode an *fHbp* variant, and these variants can be divided phylogenetically into two subfamilies, A and B. [10]. This antigen is included in the 4CMenB vaccine [11] and is the basis of the bivalent MenB-fHbp vaccine [3]. fHbp is also the antigen displayed by the novel chimpanzee adenovirus vectored MenB vaccine candidate (ChAdOx1 MenB.1) which has recently completed phase I clinical trials [12]. The replication deficient ChAdOx1 vector used in ChAdOx1 MenB.1 contains the mammalian tissue plasminogen activator (tPA) signal sequence at the N-terminus of the target protein-coding region, to ensure that the resulting antigen is addressed to the secretory pathway within the mammalian cells [13]. However, the native fHbp sequence also contains an N-terminal signal sequence, which is thought to be crucial for export of the protein to the bacterial cell membrane as well as lipidation of the lipoprotein [14], as the signal sequence contains the C-terminal lipobox motif (LTAC) (Additional file 13: Fig. S1). Signal sequences have diverse functions, affecting protein trafficking as well as marking proteins for post-translational modification [15]. What is not yet known is the effect on immunogenicity of combining mammalian signal sequences with components of native bacterial signal sequences in adenovirus vectored vaccines, specifically the impact this would have on transgene expression kinetics and how these might relate to the immunogenicity of the expressed antigen.

AdHu5 is a human adenovirus vaccine vector which, like ChAdOx1, contains the mammalian tPA signal peptide, however, a number of studies have suggested that chimpanzee adenovirus vectors are less immunogenic than AdHu5 vectors when administered intramuscularly to mice [16–21]. Thus, AdHu5 provides a versatile platform with which to investigate the effect of including different components of both native bacterial and mammalian signal sequences upstream of antigens in adenovirus vectored vaccines. Here we create a panel of fHbp N-terminal amino acid sequence variants within AdHu5 vectors for in vitro and in vivo analyses of antigen expression dynamics and vaccine immunogenicity respectively. The full-length signal sequence (FL SS) fHbp construct contains the mammalian signal peptide NRTAFCCLSLTTALI immediately upstream of the bacterial lipobox motif LTAC. Variants lacking one or both of these sequences, as well as single amino acid variants of these sequences, were assessed side-by-side to determine their relative contributions to the enhanced functional immune response. The present study focuses on the comparative assessment of SS variants using a combination of in vitro expression assays and in vivo immunogenicity data in mice to determine the link between transgene expression and the enhanced functional antibody response associated with the candidate vaccine antigen as compared with the licensed 4CMenB vaccine. In addition, the potential application of this SS to other bacterial antigens is explored with a view to developing a generalisable sequence to boost the immunogenicity of viral vectored vaccines currently undergoing pre-clinical development.

## Results

### Factor H binding protein N-terminal amino acid sequence variants induce differential functional antibody titres at early timepoints in mice

Groups of mice were immunized once with the adenoviral constructs encoding the different fHbp with N-terminal SS variants, outlined in Table 1, at a dose of 1 × 10^7^ infectious units. Dose–response studies suggest that a dose of 1 × 10^7^ infectious units per mouse, herein termed “sub-optimal”, does not induce an antibody response at plateau as higher doses would and therefore lends itself well to assessing early differences in antibody induction between the SS variants. Sera collected at several timepoints were assessed for fHbp-specific IgG titres by indirect ELISA. Statistically significant differences in anti-fHbp antibody titres were observed between signal sequence variant groups at weeks two and four post-immunisation. The highest titres were induced by the FL SS and SP KO SS constructs; the lowest titres were induced by the truncated SS, lipobox KO and 4CMenB groups (Additional file 14: Fig. S2A, B). By weeks six and 14, the only statistically significant differences observed were between 4CMenB and several signal sequence variant groups, as the response had matured to reached plateau (Additional file 14: Fig. S2C, D).

**Table 1:**
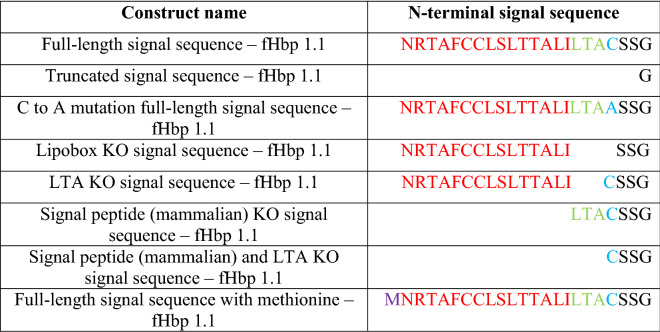
Panel of factor H binding protein signal sequence variants for comparative assessments of immunogenicity

N-terminal amino acid “signal sequences” for factor H binding protein (fHbp) 1.1 transgenes encoded by human adenovirus serotype 5 viral vector vaccines. The signal peptide portion of the sequence is highlighted in red, the LTA portion of the lipobox in green, the lipobox cysteine in blue, the N-terminal methionine in purple, and the SSG amino acids that remains following the natural cleavage of the signal peptide in black. KO, knockout.

**Fig. 1:**
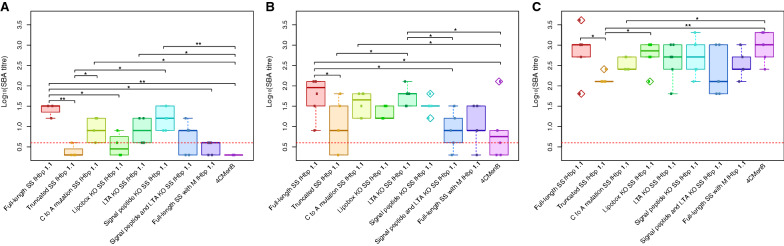
Serum bactericidal antibody (SBA) titres in sera of mice immunised with human adenovirus encoding N-terminal signal sequence variants of the factor H binding protein. Groups of six BALB/c mice were immunised with a sub-optimal dose of 1 × 10^7^ infectious units of one of the signal sequence variant constructs or 1/10 of the human dose of 4CMenB (two-dose regimen administered at day 0 and day 21) and SBA assays were performed against the H44/76 strain of *Neisseria meningitidis* using sera derived from blood samples taken at different timepoints post-immunisation. **A** Week two SBA titres. **B** Week four SBA titres. **C** Week six SBA titres. The dotted red line represents the cut-off titre of 1:4 deemed sufficient for protection. Differences in the SBA titre between constructs are attributable to the N-terminal signal sequence variants of the factor H binding protein, particularly at early timepoints post-immunisation. Statistical comparisons were made using a Mann–Whitney U-test. *p < 0.05; **p < 0.01

As MenB is known only to infect humans, suitable animal models do not exist and immune correlates are accepted as an indication of in vivo immune protection for pre-clinical assessment of vaccine candidates [22]. The SBA is the gold standard correlate of protection required for vaccine licensure. The functionality of anti-fHbp antibodies was assessed by performing SBA assays against the H44/76-SL reference strain that naturally expresses fHbp 1.1 using the sera taken at weeks two, four, and six post-immunisation. Significant differences in SBA titre were measured at each timepoint, with greatest differences observed at weeks two and four post-immunisations (Fig. 1A, B). A single dose of AdHu5 expressing the FL SS fHbp antigen induced the highest titres of bactericidal antibody at week two, significantly higher (p < 0.01) than the truncated SS fHbp, while 4CMenB failed to induce protective titres after the first dose of the two-dose regimen at this timepoint (Fig. 1A). The differences between the SBA titres associated with each construct closely resembled the differences in antigen expression levels observed after overnight infection of HeLa cells, with the exception of the lipobox (LTAC) knockout (KO) SS which was highly expressed in this assay but induced lower SBA titres at this timepoint. This trend was still apparent by week four post-immunisation, with most constructs inducing SBA titres above the threshold for protection (Fig. 1B). Low titres were induced by 4CMenB, even 1 week after the second dose of the two-dose regimen (administered at 21 days post-first dose). By week six post-immunisation, all constructs had induced SBA titres of > 1:4, the putative threshold of protection for meningococcal vaccines (Fig. 1C). At this timepoint, the truncated SS fHbp was associated with the lowest titres, significantly lower than the FL SS and lipobox KO SS fHbp variants, while similar titres were obtained from the FL SS fHbp and 4CMenB groups.

Taken together, these results demonstrate that the N-terminal SS impacts on the functional antibody titres induced by the adenoviral-driven expression of the fHbp transgene. Additionally, the FL fHbp SS is capable of inducing protective titres of SBA just two weeks after a single sub-optimal dose, while the licensed 4CMenB vaccine requires two doses and greater than four weeks to achieve such titres in mice. The pattern of bactericidal antibody response suggests that the immunogenicity of these constructs may be influenced by the expression dynamics associated with the SS variants.

### Modifying the SS ahead of the Factor H binding protein sequence impacts on the antigen expression levels at early timepoints in vitro

**Fig. 2:**
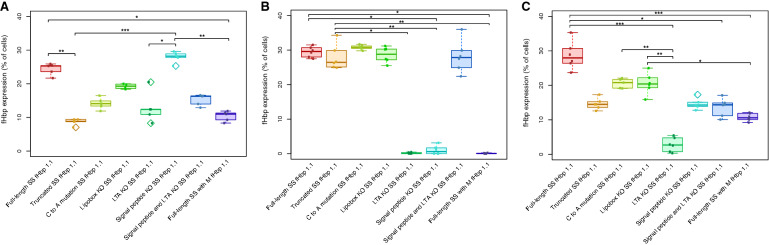
Expression of human adenovirus-encoded factor H binding protein N-terminal signal sequence variants from HeLa cells. **A** HeLa cells (1 × 10^6^ per sample) were infected overnight with 5 × 10^8^ infectious units of one of a series of human adenovirus serotype 5 (AdHu5) constructs encoding an N-terminal sequence variant of the factor H binding protein (fHbp) and expression was quantified by flow cytometry after surface and intracellular staining of harvested cells with an anti-fHbp antibody (JAR5) and a fluorescently-tagged detection antibody. Cells were tested for intracellular expression by stimulation with brefeldin A to stop protein transport within the cells and harvested at **B** three-hour and **C** five-hour timepoints post-infection to measure early expression levels by intracellular staining. The y-axis corresponds to the percentage of total fluorescent (fHbp-expressing) HeLa cells after overnight infection. The amino acid composition of the N-terminal sequence impacts upon the expression of the transgene-encoded antigen within the first 24 h of infection. Statistical comparisons were made using a Mann–Whitney U-test. *p < 0.05; **p < 0.01; ***p < 0.001

To determine whether expression kinetics underlie the differential immunogenicity of the SS variants, expression of AdHu5 vectors encoding one of each of the fHbp SS variants outlined in Table 1 was measured by flow cytometry using an anti-fHbp antibody (JAR5) and a GFP secondary antibody. 25–30% of cells infected with the signal peptide (SP) KO SS fHbp construct were fHbp positive after overnight infection, while < 10% of cells infected with the truncated SS fHbp were found to express fHbp (Fig. 2a). This implies that the lipobox (LTAC) portion of the SS is sufficient to induce the difference in early antigen expression levels observed between the FL and truncated SS fHbp constructs. The levels of expression of the lipobox KO SS construct suggests that the mammalian signal peptide does boost expression levels relative to the truncated SS fHbp, but not to the same degree as the lipobox itself. The results obtained with the LTA amino acids KO indicates that the C residue is important for this increase in expression, further evidenced by the low expression levels observed for the C to A mutation SS construct. However, knocking out both the signal peptide and the LTA portion of the lipobox results in a similarly low level of expression, highlighting the importance of an intact lipobox for boosting expression. The inclusion of a methionine (M) amino acid at the beginning of the bacterial native signal sequence, indicating a start codon at this position, also appears to negatively impact upon expression despite an otherwise unaltered SS.

By comparing the percentage of cells expressing fHbp after surface-staining only or intracellular-only staining, the intracellular expression of the antigen was shown to contribute to the majority of total antigen expression after overnight infection (Additional file 15: Fig. S3). To further examine the intracellular antigen expression levels at early timepoints, cells were treated with brefeldin A, an inhibitor that blocks protein transport to the Golgi apparatus causing protein to accumulate instead in the endoplasmic reticulum (ER), and flow cytometry was performed hourly for six hours post-infection with a single AdHu5 construct (FL SS fHbp), thus determining key timepoints for peak antigen expression (Additional file 16: Fig. S4). Based on the expression levels measured, the three-hour timepoint appears to coincide with a window of increased antigen expression while expression appears to plateau at the five-hour timepoint for this construct. These timepoints were chosen for further comparisons of early antigen expression between the mutated constructs. HeLa cells were stimulated with brefeldin A, infected with each SS variant construct, and analysed by flow cytometry for intracellular expression of fHbp between three- and five-hours post-infection (Fig. 2B). The FL SS fHbp construct was the only construct that expressed at high levels at both timepoints, while most constructs which expressed highly at three hours post-infection failed to maintain these expression levels at the five-hour timepoint (Fig. 2C). Several constructs which did not express at three hours post-infection showed modest levels of expression at the later timepoint. Surprisingly, the truncated SS fHbp construct expresses highly after three hours, but by five hours post-infection the antigen level has declined. This suggests that the SS may play a role in promoting sustained expression of antigen within cells or slows its degradation. Similar to the truncated SS fHbp, antigen levels from the other variants that express highly at three hours post-infection decline over time. The LTA KO vector induced expression at a consistently low level at each timepoint, indicating that the mammalian signal peptide portion of the SS does not appear to enhance the early expression of the antigen by itself in this mammalian cell line. The fact that the FL SS exhibits the most consistent levels of expression during these early timepoints, and the expression levels of each variant fluctuate between timepoints highlights variable contributions to the timing and persistency of antigen expression associated with each element of the entire SS.

### The amino acid composition of the N-terminal signal sequence influences the dynamics of antigen expression from HeLa cells by promoting early expression

As the in vitro expression assays alluded to differential contributions of distinct SS elements to the transgene expression kinetics, this phenomenon was further explored by selecting four SS variants—FL SS, SP KO SS, LTA KO SS, and SP + LTA KO SS—and employing microscopy techniques to visualise expression of these antigens in HeLa cells. To this end, plasmids encoding each of these antigen variants fused with eGFP via a flexible linker peptide were designed, cloned, and incorporated into AdHu5 vectors. To ensure accurate recapitulation of antigen expression, overnight expression from HeLa cells was compared between these eGFP fusion constructs and their corresponding original constructs (Additional file 17: Fig. S5). The eGFP-expressing constructs were detected in a greater proportion of infected cells, possibly due to the greater natural fluorescence intensity of these antigens compared with the detection of antibody-labelled antigens from the original constructs which may be less efficient. Overall, the trend in expression level differences was replicated for the eGFP constructs, confirming that the impact of the SS remains apparent even in the fusion antigens. The lower expression levels of the fHbp-eGFP fusion antigens compared with that of the eGFP only positive control confirms that the observed differences in expression between eGFP-containing constructs are due to the N-terminal fHbp SS variant antigens.

**Fig. 3:**
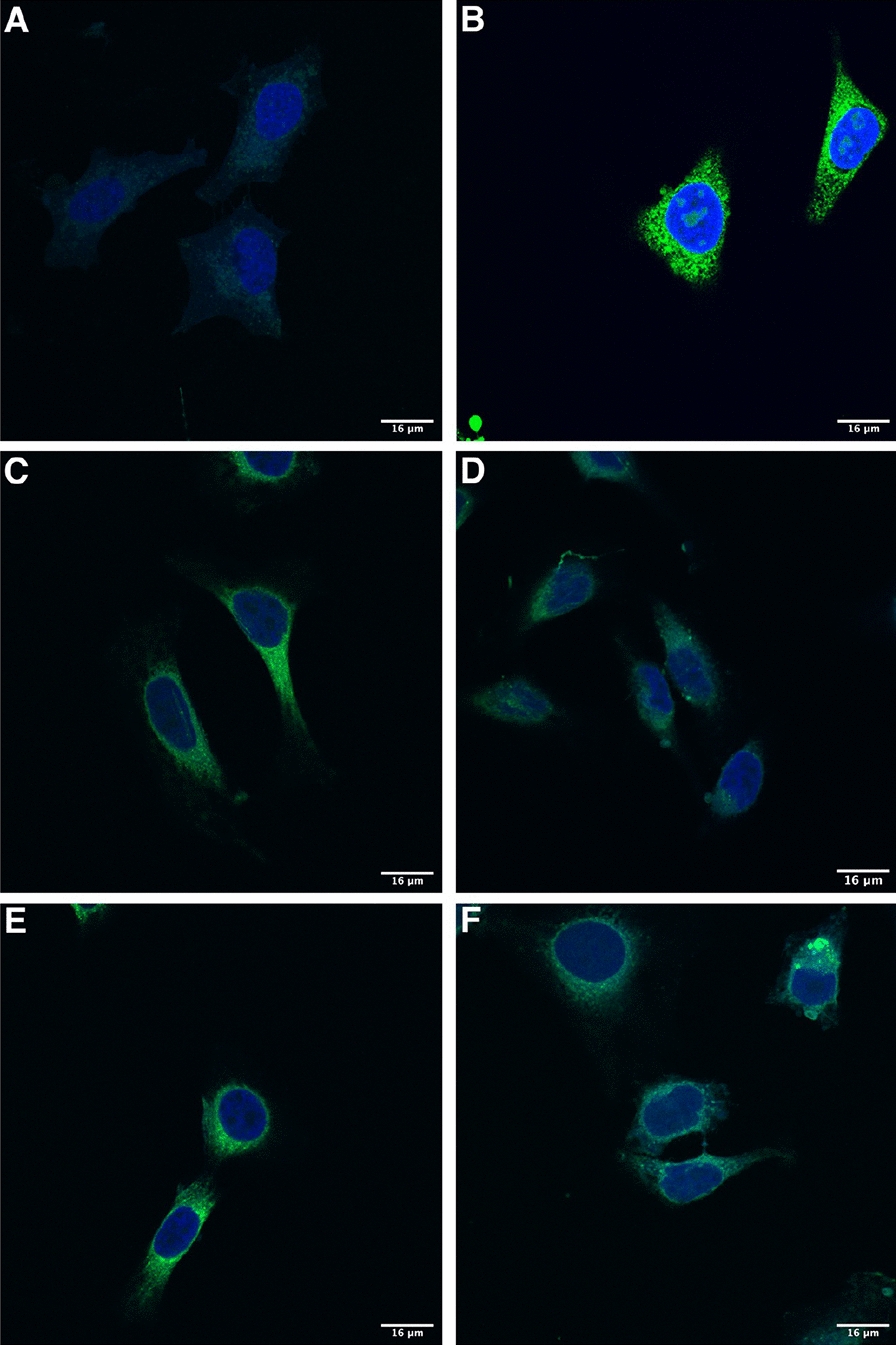
Confocal images of HeLa cells infected overnight human adenovirus encoding factor H binding protein (fHbp) N-terminal signal sequence (SS) variants. N-terminal signal sequence variant transgenes were designed to include an enhanced green fluorescent protein (eGFP) sequence and cloned into adenovirus vectors. Representative images for **A** Uninfected cells (negative control), **B** eGFP only (positive control), **C** Full-length SS fHbp 1.1-eGFP, **D** LTA knockout (KO) SS fHbp 1.1-eGFP, **E** Signal peptide (SP) KO SS fHbp 1.1-eGFP and **F** SP + LTA KO SS fHbp 1.1-eGFP. Blue fluorescence indicates DAPI-stained nuclei, green fluorescence indicates antigen-eGFP expression. 16 μm scale bars are shown in the bottom right corner of each image

Confocal microscopy was employed to visualise the expression of these fHbp-eGFP fusion antigens from AdHu5-infected HeLa cells. To relate the microscopy results to the flow cytometry data, 3 × 10^5^ cells seeded on glass coverslips placed at the bottom of six-well plates were first infected overnight with 1.5 × 10^8^ IU (to obtain a multiplicity of infection (MOI) of 500) of each eGFP-expressing AdHu5 vaccine, and then fixed, DAPI-stained, and transferred to microscope slides for imaging using a Zeiss 780 inverted confocal microscope. In accordance with the relative differences in expression levels quantified for the four constructs as measured by flow cytometry, a similar pattern was observed in the confocal microscopy images (Fig. 3). All constructs displayed a greater intensity of eGFP expression relative to a negative control of uninfected HeLas (Fig. 3A), with the eGFP only construct expressing at the greatest intensity (Fig. 3B). The FL SS and SP KO SS constructs displayed similar levels of eGFP intensity, while both the LTA KO SS and SP + LTA KO SS constructs displayed similarly low levels of eGFP intensity (Fig. 3C–F).

**Fig. 4:**
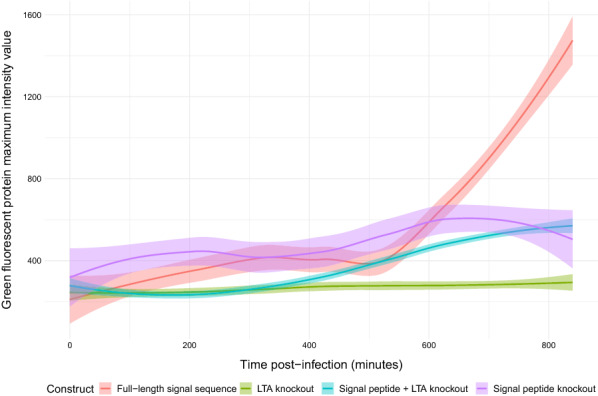
Expression of factor H binding protein N-terminal signal sequence variants from HeLa cells infected with adenovirus vectors over the course of 14 h. N-terminal signal sequence variant transgenes were designed to include an enhanced green fluorescent protein sequence and cloned into adenovirus vectors. Green fluorescent protein intensity values were calculated from images taken across three separate fields of the infected wells and averaged for each signal sequence variant. Loess smoothing was applied to trend lines for each construct and 95% confidence intervals are shaded

To compare the expression dynamics of these fluorescent antigen variants in a longitudinal manner, 1 × 10^5^ HeLa cells were seeded overnight in each well of an eight-well chambered coverslip. The following day the cells were stained using far-red fluorogenic SiR-DNA, infected with 5 × 10^7^ IU (to obtain an MOI of 500) of each eGFP-expressing AdHu5 vaccine, and imaged every ten minutes over the course of 14 h using a Zeiss Spinning Disc microscope. Time-lapse images for each infection were obtained across three separate fields for each well of AdHu5-infected cells (Fig. 4, Additional files 1, 2, 3, 4, 5, 6, 7, 8, 9, 10, 11, 12). Differences were observed between the timings of antigen expression between the constructs, particularly at early timepoints post-infection. The FL and SP KO SS variants displayed similar antigen expression kinetics. The SP + LTA KO SS variant was expressed at a marginally later time and at a lower level, while the LTA KO resulted in the lowest levels of antigen expression. These images confirm the in vitro antigen expression data and provide further evidence for the differential contribution of these specific SS elements to early antigen expression from mammalian cells.

### Inclusion of the full-length signal sequence at the N-terminus of other adenovirus-encoded bacterial antigens boosts antigen-specific T cell responses to the transgene product

**Fig. 5:**
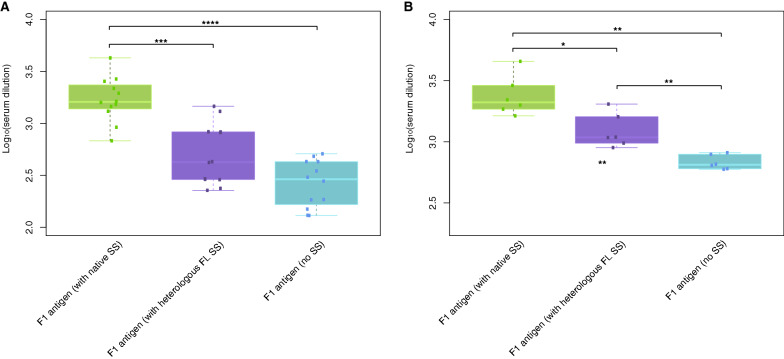
Anti-F1 antigen IgG titres in mouse sera post-immunisation with human adenovirus vectors encoding signal sequence variants of the F1 antigen. Groups of 12 BALB/c mice were immunised with a sub-optimal dose of 1 × 10^7^ infectious units of one of the human adenovirus serotype 5 (AdHu5) vectors encoding the *Yersinia pestis* F1 antigen with native signal sequence (SS), a heterologous N-terminal full-length (FL) SS, or a truncated form of the F1 antigen lacking any SS. Enzyme-linked immunosorbent assays were performed on serum samples taken at weeks **A** two and **B** four post-immunisation to determine the titres of anti-F1 antigen IgG in sera. The humoral response induced by the F1 antigen with native SS was superior to that induced by the incorporation of the heterologous FL SS to this antigen or its truncated form at both timepoints. Statistical comparisons were made using a Mann–Whitney U-test. *p < 0.05; **p < 0.01; ***p < 0.001; ****p < 0.0001

Given that the FL SS induced the most consistent protective antibody responses across timepoints for the fHbp antigen, this SS was chosen to determine whether it could be applied to other bacterial antigens to increase immunogenicity as a generalisable transgene sequence element. This was first tested for an infection that relies predominantly on humoral immune responses for protection, *Yersinia pestis* (*Y. pestis*)*.* Vectors incorporating antigens from *Y. pestis*, the causative organism of the disease plague, were constructed. The plasmids encoded the F1 antigen with its native SS, or lacking its native SS, or replacing the native SS with the FL fHbp SS. Groups of 12 BALB/c mice were immunised with 1 × 10^7^ IU of one of each of the AdHu5-plague vaccines. The anti-F1 IgG titres were measured from serum samples taken at weeks two and four post-immunisation by an indirect ELISA. The inclusion of the heterologous FL SS significantly increases anti-F1 IgG titres compared with the AdHu5 F1 construct at week four post-immunisation, though the titres were still significantly lower than those associated with the F1 antigen with native SS (Fig. 5).

**Fig. 6:**
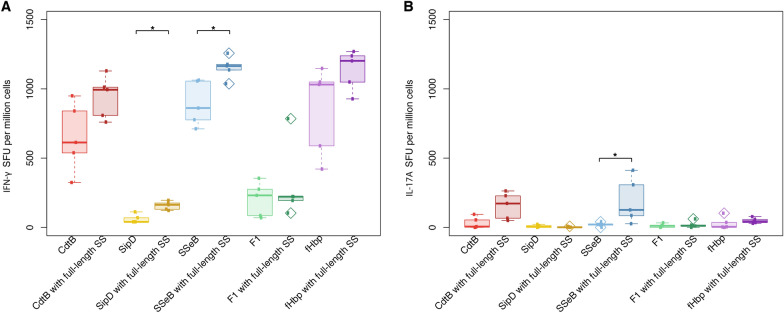
Antigen-specific T cell responses induced in mice two weeks after immunisation with human adenovirus vectors encoding bacterial antigens with or without an N-terminal signal sequence. Groups of six BALB/c mice were immunised with human adenovirus serotype 5 (AdHu5) vaccines expressing one of a series of bacterial antigens, with or without an N-terminal signal sequence (SS). Spleens were harvested two weeks post-immunisation, processed, and stimulated at a concentration of 3 μg/mL with the relevant peptide pool. An interferon (IFN)-γ/interleukin (IL)-17A dual colour fluorospot was performed to assess antigen-specific T cell responses associated with these cytokines. **A** IFN-γ and **B** IL-17A spot-forming units (SFU) per million cells were quantified for each antigen. Inclusion of an N-terminal SS was found to boost both types of antigen-specific T cell responses. Statistical comparisons were made using a Mann–Whitney U-test. *p < 0.05

To determine whether a more robust cellular response could be induced against bacterial antigens that utilise a signal peptide for expression in their native state by incorporating the generalisable FL SS ahead of the antigen sequences, antigen-specific T cell responses were assessed for the *Y. pestis* F1, MenB fHbp, and several *Salmonella* antigens—CdtB, SipD, and SseB. Plasmids were designed with the native sequence or dual SS ahead of each of these antigens, cloned, and incorporated into AdHu5 vectors. Groups of six BALB/c mice were immunised with 1 × 10^7^ IU of each AdHu5 vaccine encoding each of the bacterial antigens with or without the N-terminal SS. Spleens were harvested two weeks post-immunisation to assess antigen-specific IFN-γ- and IL-17A-producing T cell responses by fluorospot. Inclusion of the N-terminal SS boosted the antigen-specific IFN-γ-producing T cell responses to three of the five antigens tested, particularly for SipD and SseB (Fig. 6A), and even led to a significant increase in SseB-specific IL-17A-producing T cells which were otherwise mostly undetectable against the native counterparts of each antigen (Fig. 6B). These data highlight the additional attribute of the N-terminal SS in boosting antigen-specific T cell responses against heterologous bacterial antigens and demonstrate the utility of the SS as a broadly-applicable immune-enhancing peptide.

## Discussion

This study provides a functional exploration of the distinct elements of a novel signal sequence included ahead of a MenB lipoprotein expressed from a viral vector-encoded transgene. The relative contribution of each of these elements was assessed through expression assays in a mammalian cell line and validated with the use of high-resolution microscopy. The elucidation of the transgene expression kinetics provides a potential link with differences observed between these constructs through comparative immunogenicity analyses. Furthermore, the inclusion of the SS ahead of several other transgenes encoding heterologous bacterial antigens demonstrates the utility of this design in promoting early antigen-specific cellular immune responses.

Transgene antigen expression kinetics play an important role in determining the magnitude and quality of the immune response to adenovirus-encoded antigens. Several strategies have been applied to optimise transgene expression through modification of the transgene sequence, promoting the expression and cellular processing of the antigen. Most antigens that are destined for secretion or translocation to the membrane contain SPs after translation [15] and their inclusion may promote favourable processing of vaccine antigens. There is a paucity of data on the expression of bacterial antigens from mammalian cells, with the exception of the *Mycobacterium tuberculosis* (*Mtb*) Ag85B-TB10.4 antigen [13]. This mechanism has however been successfully exploited for a number of viral and parasitic antigens. For example, the cleavage of the HIV-1 envelope protein (Env) SP has been correlated with the rate of transport and folding of its constituent glycoprotein gp120 within the ER of the host cell [23]. The inclusion of the SP alters the pattern of glycosylation of this antigen and the addressing of the peptide to the secretory pathway which in turn influences the antigenic properties of gp120, a phenomenon that may prove useful from the perspective of HIV vaccine immunogen design [24]. Certain antigens have stronger SPs than others and therefore lend themselves to the enhancement of heterologous antigen processing. The inclusion of a SP from VSV has been shown to enhance the generation of Japanese encephalitis virus (JEV) envelope protein-based lentivirus vectors to a greater degree than the native JEV envelope SS [25]. A plant-derived SS has been used to elicit stronger antibody and cellular responses directed at human papillomavirus (HPV) antigens delivered via DNA vaccines to mice [26]. The use of host-optimised SPs has been met with some success; the mouse-derived IgG kappa light chain SP has been shown to significantly increase the immunogenicity of a simian adenovirus-encoded *Plasmodium vivax* antigen in mice [27]. Leader sequences have also been incorporated into viral vectors and have demonstrated their ability to boost transgene antigen-specific humoral and cellular responses. A prime example is the human tissue plasminogen activator (tPA) sequence that boosts these immune responses when placed before the *Mtb* Ag85B-TB10.4 antigen encoded by a modified vaccinia Ankara vector [13]. Modification of bacterial proteins with lipid moieties is known to facilitate the attachment of these proteins to lipid membranes, cellular transport, and folding outside of the cytoplasm [28]. The precursor of fHbp contains a lipobox element (in this case LTAC) at the end of the bacterial SP and is modified at the invariant C residue for subsequently cleavage of the SP by signal peptidases at this site, leaving C as the N-terminal amino acid [14]. A mammalian SP was selected to promote expression of the lipoprotein within mammalian tissues. The results of the expression assays presented here confirm that the FL SS is superior to the truncated SS for the promotion of fHbp 1.1 antigen expression in mammalian cells. It has also been demonstrated that these SS elements were judiciously chosen for their combined influence on transgene expression and immunogenicity; the expression assay data provides rationale for the inclusion of the FL SS within the vaccine antigen sequence as the FL sequence promotes the most consistent levels of early transgene expression across each of the chosen timepoints.

The application of confocal and time-lapse microscopy was more revealing as to the timing and intracellular localisation of antigen expression in a mammalian cell line and confirmed the findings of the in vitro expression assays. As was shown by immunofluorescence analysis of the *Phaseolus vulgaris* SS-HPV antigen expression from DNA vaccines in infected HEK293 cells, fHbp-eGFP transgene expression was predominantly located within the cytoplasm in apparent association with membrane structures and no fluorescence detected in the extracellular media [26]. This is in accordance with the typical role of SSs in targeting antigens to the membranes of the ER and cell surface [29], and studies of secretory protein trafficking in yeast have provided evidence that slight variations in the amino acid composition of SSs can result in altered recognition of these proteins by ER translocons [30]. Further to this, certain SSs can impact on the post-ER translocation of antigens; the processing of the HBV core preprotein SS results in accumulation of the cleaved, translocated product within the cytosol [31]. This may explain the differential accumulation of the SS variants following their expression in HeLa cells.

Delivery of exogeneous antigen via viral vectored vaccines has been explored for the prevention of a wide range of diseases, particularly those requiring strong CD8^+^ T cell responses. This vaccine platform also has strong potential to induce robust humoral responses, making them an attractive target for diseases requiring antibody-mediated protection. Invasive meningococcal disease is one such disease, and vaccines have been licensed on the basis of their ability to induce a human SBA titre of ≥ 4, the putative correlate of protection against the disease [32]. All fHbp 1.1 SS variants elicited protective titres of antibody in mice within four weeks of a sub-optimal dose, while the FL SS fHbp 1.1 induces protective titres by as early as week two post-immunisation. This in contrast to 4CMenB which, at 1/10 of the human dose, requires two doses and more than four weeks to induce similar titres in mice. The FL SS also demonstrates superiority to the truncated SS variant, demonstrating its ability to enhance the antigenicity of the fHbp antigen. The level of antigen expression in APCs has been highlighted as an important contributing factor to the formation of antibodies against the transgene product from adenovirus vectors [33]. The induction of antigen-specific humoral immunity is primarily mediated by the interaction of CD4^+^ T_H_ cells with MHC class II-presented peptides on the surface of these cells. The subset of CD4^+^ T_H_ cells can influence the nature of the humoral response; IFN-γ production is a hallmark of T_H_1 cells that promote IgG2 and IgG3 production from B cells [34]. Both of these antibodies are important in the context of early childhood as this age group is known to be poor at inducing these antibody subclasses, with the former playing an important role in immunity to *Neisseria meningitidis* [35]. IL-17A production indicates T_H_17 cell activity, the role of which is of growing interest to vaccinologists due to its association with vaccine-induced immune responses against a variety of bacterial pathogens, such as *Bacillus pertussis* and *Streptococcus pneumoniae* [36]. The ability of the FL SS to enhance these distinct CD4^+^ T cell responses against the neisserial fHbp, the plague F1 antigen, and the *Salmonella* antigens CdtB, SseB, and SipD was investigated and found to significantly increase some of these responses. The increase in T_H_1-mediated antigen-specific responses against SipD and SseB were significant, as was the T_H_17-mediated response against SseB. Both of these proteins are constituents of the *Salmonella* type III secretion system (T3SS) and their potential as candidate vaccine antigens has been highlighted by studies in mice and humans [37, 38]. CD4^+^ T cells of the IFN-γ and IL-17-producing variety also contributed to the response induced by the live-attenuated *Salmonella* Typhi vaccine, Ty21a [39], in humans. The findings of the antigen-specific T cell responses provide compelling evidence that the FL SS can be incorporated ahead of adenovirus-encoded heterologous bacterial antigen transgene sequences to boost antigen-specific immune responses. This has important implications for the design of candidate viral vectored vaccines against intracellular bacteria, like *Salmonella*, which require predominantly T cell-mediated immune responses [40].

There are two major limitations to consider when assessing the suitability of the Ad-fHbp vaccine for human applications. The first is the lack of a suitable animal model to assess the pre-clinical efficacy of the vaccine; a mouse challenge model does not exist for meningococcal disease as *Neisseria meningitidis* is an obligate human pathogen. Nevertheless, the SBA has proven to be a reliable indication of in vivo protection for vaccine licensure and remains the gold standard correlate of protection for MenB vaccine candidates [22]. Secondly, adenovirus vaccines used as viral vectors for COVID-19 have been reported to trigger vaccine-induced thrombotic thrombocytopenia (VITT) [22], an extremely rare complication that is thought to involve platelets, the endothelium and the blood coagulation system. An important consideration is whether such events are associated with other adenovirus vectored vaccines.

## Materials and methods

### Animal procedures

All procedures were performed in accordance with the terms of the UK Home Office Animals Act Project License. Procedures were approved by the University of Oxford Animal Care and Ethical Review Committee. The study was carried out in compliance with the ARRIVE guidelines [41]. General anaesthesia was induced using 3.5% isofluorane mixed with 2 L/min of O_2_ released into the mouse anaesthetic chamber and then via direct inhalation for each mouse through a tube while procedures were being performed. Cardiac bleeds (followed by cervical dislocation), immunisations and terminal bleeds were performed under general anaesthesia. All mice were female BALB/c (Charles River) and aged between six- and eight-weeks of age at the beginning of each experiment. Once anaesthetised, vaccines were administered to the mouse by injecting no more than 50 μL into each of the musculus tibialis at the back of the leg, using a 29-gauge insulin syringe. These volume constraints for intramuscular injection prevented the use of the 2/5 human dose tested in the original 4CMenB pre-clinical studies published by Giuliani et al. [42] by this route. The reactogenicity induced by this dose administered via the intraperitoneal route was too severe to continue with the experiment and may not have recapitulated the responses induced by this vaccine when administered intramuscularly. Therefore, 1/10 of the human dose of 4CMenB was determined the appropriate dose for intramuscular injection in the present study. 4CMenB (Bexsero®, GlaxoSmithKline) was administered as a two-dose regimen at day 0 and day 21. All other vaccines were administered as single doses on day 0. Tail bleeds were performed to obtain blood samples prior to terminal bleeds. Using a 37 °C heating box, mice were pre-warmed and restrained to facilitate nicking of the tail vein to collect 8–10 drops of blood in a 2 mL tube. For terminal bleeds, mice were put under general anaesthetic and cardiac bleeds were performed followed by cervical dislocation to confirm death.

### Splenocyte isolation

Spleens were removed by incising the left-hand side of the abdomen, after cardiac bleed and cervical dislocation of the animal, and transferred to gentleMACs™ C tubes (Miltenyi Biotec) containing autoMACS® running buffer (Miltenyi Biotec). Spleens were macerated in a gentleMACS™ Dissociator (Miltenyi Biotec) using the mouse spleen pre-set program and then tubes were centrifuged at 250 × *g* for 10 min. The supernatants were discarded, and pellets resuspended in red blood cell lysis solution for 5 min. Sterile PBS was added to quench the lysis solution after 5 min and the cell suspensions were passed through 70 um filter into 50 mL Falcon tubes using Pasteur pipettes. The filtered solutions were centrifuged again at 250 ×*g* for 10 min, supernatants discarded, and pellets resuspend in 10 mL of complete DMEM. A Muse® Count & Viability Assay Kit (Millipore) was used to measure the final cell count and viability of each sample on a Muse® Cell Analyzer instrument (Millipore). Samples were diluted to 4 × 10^6^ in complete DMEM.

### Plasmid construct design

The nucleotide sequence for the desired antigen was obtained from the GenBank sequence database (https://www.ncbi.nlm.nih.gov/genbank/). The sequence was run through a glycosylation site finder (http://www.cbs.dtu.dk/services/NetNGlyc/) to identify putative glycosylation sites and remove them if the probability of glycosylation exceeds 50%. This is done to remove sequences that may be post-translationally modified if expressed from mammalian cells. The polyA tail was removed from the sequence and the GeneArt® Gene Synthesis tool (Invitrogen) (https://www.thermofisher.com/en/home/life-science/cloning/gene-synthesis/geneart-gene-synthesis.html) was used to upload the sequence, optimise codons for mammalian tissue, and add restriction sites. The HindIII (AAGCTT at the start of the sequence) and NotI (GCGGCCGC at the end of the sequence) restriction sites were added to the construct.

### Restriction digest

To cut out the transgene from the plasmid backbone, a restriction digest reaction was set up as a 50 μL reaction in a PCR tube as follows:

**Table:** 

Reagent	Volume/concentration
Restriction enzyme (HindIII-HF, NotI-HF, New England Biolabs)	1 μL of each
10X CutSmart® buffer (New England Biolabs)	5 μL
Plasmid DNA	1 μg
dH_2_O	Remaining volume (up to 50 μL)

The reaction was run for four hours at 37 °C on a thermocycler

### Agarose gel electrophoresis

DNA samples were run on a 1% agarose gel after restriction digest. The gel was stained using peqGreen DNA dye and placed in an electrophoresis cassette filled with 1X TAE buffer. 10 μL of purple loading dye was added to the 50 μL samples to achieve a 1X concentration. Samples were loaded into the wells of the gel along with a 1 kb DNA ladder. The electrophoresis apparatus was connected to a voltage box set to 100 V and the gel was run for 40 min. The gel was analysed using a UV light box and bands corresponding to the size of the insert were excised from the gel using a gel extractor tool and placed in a 2 mL Eppendorf tube. DNA was extracted from the gel section using a QIAquick Gel Extraction Kit (QIAGEN). Briefly, kit buffer was added to the 2 mL tube at a ratio of 3:1 (100 mg gel ~ 100 μL) and incubated at 50 °C on a heating block for 10 min or until dissolved, one volume of isopropanol was added and the gel digest was filtered and washed as per kit instructions, eluting the DNA into a final volume of 100 μL dH_2_O. The DNA concentration was measured using a NanoDrop™ 2000 Spectrophotometer (ThermoFisher Scientific).

### DNA ligation

A 20 μL DNA ligation reaction was set up, to place the insert sequence into a pMONO expression plasmid with Kanomycin resistance, in a PCR tube as follows:

**Table:** 

Reagent	Volume/concentration
2X LigaFast™ buffer (New England Biolabs)	5 μL
pMONO 164 backbone DNA	2 μL
Insert DNA	2 μL
T4 DNA ligase (New England Biolabs)	1 μL

The reaction was run overnight on a thermocycler set to 16 °C.

### Transformation

After overnight ligation, 5 μL of sample was added to 50 μL of thermocompetent DH5α cells (ThermoFisher Scientific) in a 2 mL Eppendorf tube on ice. A negative control was set up by adding 5 μL of dH_2_O to the same volume of cells. Samples were incubated at 4 °C for 30 min, heat-shocked at 42 °C for 30 s, and returned to 4 °C for 2 min. 250 μL of pre-warmed (37 °C) SOC recovery medium (New England Biolabs) was added to each tube and samples were placed on a 37 °C shaking incubator for 1 h before plating 200 μL on LB agar containing 30 μg/mL Kanomycin using an L-shaped spreader. Plates were incubated at 37 °C overnight. The following day, colonies were picked from the plates using a pipette tip and dropped in an Erlenmeyer shaker flask containing 50 mL of LB broth with 30 μg/mL Kanomycin to be placed in a 37 °C shaking incubator overnight.

### DNA extraction

Samples were transferred from the Erlenmeyer flasks for 50 mL Falcon tubes and centrifuged at 300 ×*g* for 10 min. The supernatant was poured off and DNA was extracted from the pellets using a QIAGEN Plasmid Midi Kit, as per manufacturer’s instructions. DNA concentrations were measured using the NanoDrop™ and subsequently restriction digested. Aliquots of the digested DNA samples were run on a 1% gel and, if the band corresponded to the appropriate insert size, sent to Source Bioscience for sequencing, using forward and reverse primers for the 3′ cytomegalovirus (CMV) promoter and bovine growth hormone (bGH) polyA region sequences, respectively, to ensure the presence of the correct antigen sequence in these samples.

### Cloning into adenovirus

Sample sequence files, forward and reverse, were aligned to the plasmid containing the transgene insert reference file using SeqMan Pro (DNASTAR) software. For samples with 100% sequence identity to the reference, DNA was treated with a Gateway™ LR Clonase™ II kit. The DNA samples, containing the antigen sequence of interest, were diluted in TE buffer to yield a solution containing 150 ng of DNA which was then combined with 1 μL of an AdHu5 destination vector (at a concentration of 150 ng/μL) in a PCR tube. 2 μL of LR Clonase™ II Enzyme Mix was added to each sample and incubated at room temperature for 10 min. 1 μL of proteinase K was added to each sample the following day and incubated at 37 °C for 10 min. The proteinase K-digested samples were transformed using DH5α by plating 200 μL on LB agar containing 100 µg/mL ampicillin and adding 10 μL streaks of up to four samples per plate of LB agar containing 15 µg/mL chloramphenicol. Plates were incubated overnight and the following day samples with colonies on LB ampicillin plates that did not grow on LP chloramphenicol plates were picked and cultured overnight in a 37 °C shaking incubator in Erlenmeyer flasks containing 50 mL of LB broth with 100 µg/mL ampicillin. DNA was extracted the following day, the concentration was measured, and samples were again sent for sequencing with Source BioScience using the same primers. Samples with 100% sequence identity relative to a reference sequence (AdHu5 destination vector containing the antigen insert) were selected for linearization. 1 µg of DNA was restricted by 3 µL of PacI restriction enzyme in 10 µL of CutSmart® buffer (New England Biolabs) and made up to 100 µL total volume with dH_2_O in a PCR tube. The reaction was run for four hours at 37 °C on a thermocycler and the reaction was stopped by exposing to 65 °C for 25 min. 15 µL of sample was run on an agarose gel to ensure linearization was successful and the remaining volume (85 µL) was sent to the Viral Vector Core Facility (VVCF, Jenner Institute, University of Oxford) for production.

### Expression assays

Cells were harvested at approximately 70–80% confluency. Where brefeldin stimulation was required, cells were resuspended in media containing 3 μg/mL of brefeldin A solution (ThermoFisher Scientific). 1 × 10^6^ cells per sample were infected with the appropriate volume of adenovirus construct to obtain a concentration of 5 × 10^8^. Infected cells were incubated for the required time (overnight or specific time points), harvested and surface stained and/or stained intracellularly with anti-fHbp monoclonal antibody JAR5 (National Institute of Biological Standards and Controls) and GFP-tagged IgG detection antibody using a Fixation/Permeabilization Solution Kit. Cells were resuspended in 400 μL of permeabilization buffer and filtered before running on a FACSCalibur flow cytometer (BD Biosciences). The percentage of cells expressing GFP was measured and analysis was performed using FloJo software (TreeStar).

### Indirect enzyme-linked immunosorbent assays

To quantify antibodies against fHbp in animal sera, 2.5 μg/mL recombinant fHbp 1.1 (plasmid gifted by Dr Peter Beernink, CHORI, and protein produced at Oxford Protein Production Facility) in carb/bicarb buffer was coated onto 96-well flat-bottomed microtitre plates overnight at 4 °C. Plates were washed with PBS containing 0.05% (v/v) Tween-20. Plates were blocked with 1% BSA in PBS and serial dilutions of sera or positive control (JAR5 antibody) were added. After incubation, anti-goat anti-mouse horseradish peroxidase conjugate was added at a 1:20,000 dilution. Plates were incubated for 20 min with 100 μL per well TMB enzyme-linked immunosorbent assay (ELISA) substrate. 1 M sulphuric acid was added to stop the reaction. Absorbance were read at 450 nm and 630 nm using a Multiskan MS plate reader (Biotek). Interpolated IgG concentrations were calculated for each serum sample using a standard curve.

### Serum bactericidal antibody assays

H44/76-SL (provided by Public Health England, PHE) strain *Neisseria meningitidis* group B bacteria were used for SBAs. Test sera (20 μL) were heated for 30 min at 56 °C to inactivate endogenous complement. Equal volumes (10 μL) of bacterial suspension (optical density (OD)600 = 0.1 bugs diluted to 1:5000), and human complement were added to two-fold serial dilutions of test serum sample. The SBA titre was the serum dilution resulting in 50% survival of the bacteria compared to the number of colony-forming units (CFU)/mL in the variable complement control, containing inactivated complement and no sera.

### FluoroSpot assays

A Mouse IFN-γ/IL-17A FluoroSpot kit (Mabtech, FSP-4144–10) was used to measure antigen-specific T cell responses in mouse spleen tissue. Anti-IFN-γ and anti-IL-17A monoclonal capture antibodies were diluted in PBS to a concentration of 15 μg/ml and 10 μg/ml, respectively. The IPFL plate membrane was washed with 15 μL of 35% ethanol per well for no more than 60 s and then washed five times with 200 μL of sterile H_2_O per well. 100 μL of capture antibody was added to each well and the plate was sealed for overnight incubation at 4 °C. The plate was washed five times with 200 μL of sterile PBS per well the following day and the wells were then blocked with DMEM containing 10% FBS for 30 min at room temperature. Stimuli, either DMSO (1:100) as a negative control, ConA (12 μg/mL) as a positive control, or reconstituted peptide pools (3 μg/mL) corresponding to the vaccine antigen, were added to the appropriate wells, followed by 2 × 10^5^ splenocytes from the appropriate sample to each corresponding well. The plate was sealed and incubated overnight at 37 °C in a 5% CO_2_ incubator. Cells were removed from the wells the following day and the plate was washed five times with 200 μL of PBS per well. Anti-IFN-γ-R4-6A2-BAM and anti-IL-17A-MT2270 (biotinylated) detection antibodies were diluted in PBS containing 0.1% BSA in the same tube to a concentration of 1:200 and 1:250, respectively. 100 μL of detection antibody mixture was added to each well and incubated for two hours at room temperature. Anti-BAM-490 and SA-550 fluorophore conjugates were both diluted to a concentration of 1:200 in the same tube with PBS containing 0.1% BSA and, after washing the plate five times with PBS, 100 μL of this mixture was added to each well. The plate was wrapped in aluminium foil and incubated for one hour in the dark at room temperature. The plate was washed five times with PBS before adding 50 μL of fluorescence enhancer to each well and incubating in the same manner for 15 min. The plate was emptied of all liquid and the underdrain was removed. Plates were completely dried in the dark at room temperature prior to spot counting with AID ELISpot software 8.0 (Autoimmune Diagnostika). Excitation 490 nm/emission 510 nm (FITC) and excitation 550 nm/emission 570 nm (Cy3) wavelengths were used to measure IFN-γ and IL-17A, respectively.

### Confocal microscopy

Complete DMEM containing 1 × 10^6^ HeLa cells were seeded in 300 μL volumes per well on glass coverslips placed at the bottom of six-well plates. Cells were infected overnight with 1.5 × 10^8^ IU (proportional to the number of cells infected) of each AdHu5 vaccine containing an antigen-eGFP transgene or appropriate control. Cells were washed the following day using sterile PBS and fixed and permeabilised using 250 μL of Fixation/Permeabilization solution from and incubated at 4 °C for 20 min. After washing with PBS, 300 μL of 300 nM DAPI (5 mg/mL stock solution diluted to 300 nM in PBS) was added to the fixed/permeabilised cells for 5 min at 4 °C, washed again and slowly transferred, using forceps and dabbing off excess liquid, to microscope slides with a drop of ProLong Gold Antifade Mountant in the centre. Slides were cured on a flat surface overnight in the dark and imaged the following day using a Zeiss 780 inverted confocal microscope (Zeiss) with ZEN Lite image acquisition software (Zeiss). Cells were located in brightfield and then the interface was switched to ‘acquisition mode’ where ‘smart settings’ were applied. The fluorophore-specific settings were manually refined, and images were taken across multiple planes of focus for each sample triplicate.

### Time-lapse imaging

Complete DMEM containing 1 × 10^6^ HeLa cells were seeded in 100 μL volumes per well (1 × 10^5^ cells per well) overnight in each well of an eight-well chambered coverslip. The following day the cells were stained using far-red fluorogenic SiR-DNA 2 h prior to infection. Cells were washed with PBS and subsequently infected with 5 × 10^7^ IU (proportional to the number of cells) of each eGFP-expressing AdHu5 vaccine. The chambered coverslip was then secured on the stage, within a live cell stage incubator set to 37 °C and supplemented with 5% CO_2,_ of a ZEISS Spinning Disc microscope (Zeiss). Three coordinates were set for each sample using ZEN Blue image acquisition software (Zeiss) and imaged every 10 min over the course of 14 h. The time-lapse for each sample was then constructed from these images using ImageJ software (Fiji).

## Supplementary Information

**Additional file 1: **FL_SS_1.

**Additional file 2: **FL_SS_2.

**Additional file 3: **FL_SS_3.

**Additional file 4: **LTA_KO_1.

**Additional file 5: **LTA_KO_2.

**Additional file 6: **LTA_KO_3.

**Additional file 7: **SP_KO_1.

**Additional file 8: **SP_KO_2.

**Additional file 9: **SP_KO_3.

**Additional file 10: **SP_LTA_KO_1.

**Additional file 11: **SP_LTA_KO_2.

**Additional file 12: **SP_LTA_KO_3.

**Additional file 13: Figure S1.** Schematic of the N-terminal full-length signal sequence upstream of the factor H binding protein lipoprotein sequence.

**Additional file 14: Figure S2.** Antigen-specific IgG titres in sera from mice immunised with human adenovirus vectors encoding N-terminal sequence variants of the factor H binding protein antigen. Groups of six BALB/c mice were immunised with a sub-optimal dose of 1 × 10^7^ infectious units of one of the human adenovirus serotype 5 (AdHu5) vectors encoding factor H binding protein (fHbp) with N-terminal signal sequence variants or 1/10 of the human dose of 4CmenB as a comparator. Enzyme-linked immunosorbent assays were performed on serum samples taken at weeks **(A)** two, **(B)** four, **(C)** six, and **(D)** 14 post-immunisation to determine the titres of anti-fHbp IgG in sera. Statistical comparisons were made using a Mann-Whitney U-test. * p < 0.05; ** p < 0.01; *** p < 0.001.

**Additional file 15: Figure S3.** Intracellular and surface expression of human adenovirus-encoded factor H binding protein with a full-length N terminal signal sequence on HeLa cells after overnight infection. HeLa cells (1 × 10^6^ per sample) were infected overnight with 5 × 10^8^ infectious units of a human adenovirus serotype 5 (AdHu5) construct encoding the factor H binding protein (fHbp) with a full-length signal sequence and expression was quantified by flow cytometry after surface only, intracellular only, or both surface and intracellular staining of harvested cells with an anti-fHbp antibody (JAR5) and a fluorescently-tagged detection antibody. The y-axis corresponds to the percentage of total fluorescent (fHbp-expressing) HeLa cells after overnight infection. The vast majority of antigen is expressed within the cell at this timepoint.

**Additional file16: Figure S4**. Time-course expression assay of human-adenovirus-encoded factor H binding protein with a full-length N-terminal signal sequence on HeLa cells stimulated with brefeldin. A. HeLa cells (1 × 10^6^ per sample) were stimulated with brefeldin A to stop protein transport within the cells and subsequently infected with 5 × 10^8^ infectious units of a human adenovirus serotype 5 (AdHu5) construct encoding the factor H binding protein (fHbp) with a full-length signal sequence and expression was quantified by flow cytometry after intracellular only staining of harvested cells with an anti-fHbp antibody (JAR5) and a fluorescently-tagged detection antibody. The y-axis corresponds to the percentage of total fluorescent (fHbp-expressing) HeLa cells after each hourly timepoint post-infection (x-axis) up to six hours. The antigen begins to be expressed at high levels at three hours post-infection and expression levels plateau around five hours.

**Additional file17: Figure S5.** Side-by-side comparison of factor H binding protein N-terminal signal sequence variants, with and without enhanced green fluorescent protein tags, expressed from human adenovirus vectors after overnight infection HeLa cells. HeLa cells (1 × 10^6^ per sample) were infected overnight with 5 × 10^8^ infectious units of one of a series of human adenovirus serotype 5 (AdHu5) constructs encoding an N-terminal sequence variant of the factor H binding protein (fHbp), fHbp fused with enhanced green fluorescent protein (eGFP), or GFP only, and expression was quantified by flow cytometry after surface and intracellular staining of non-GFP-expressing cells with an anti-fHbp antibody (JAR5) and a fluorescently-tagged detection antibody. The y-axis corresponds to the percentage of total fluorescent (fHbp- and/or GFP-expressing) HeLa cells after overnight infection. The differences in expression levels between the antigen variants tested is conserved for the eGFP fusion antigens and lower for the eGFP fusion antigens than the eGFP only positive control, confirming that the differences in antigen expression levels are attributable to the fHbp N-terminal signal sequence.

## Data Availability

https://idr.openmicroscopy.org/webclient/?show=screen-1151 – will be deposited pending acceptance of the manuscript for publication. All other data can be made available by the corresponding author upon reasonable request.
