# Supplementary figures and images for: Inclusion of a dual signal sequence enhances the immunogenicity of a novel viral vectored vaccine against the capsular group B meningococcus
Source: Cell Biosci. 2022 Jun 11;12:86. doi: 10.1186/s13578-022-00809-3 (PMC9187930; doi:10.1186/s13578-022-00809-3)

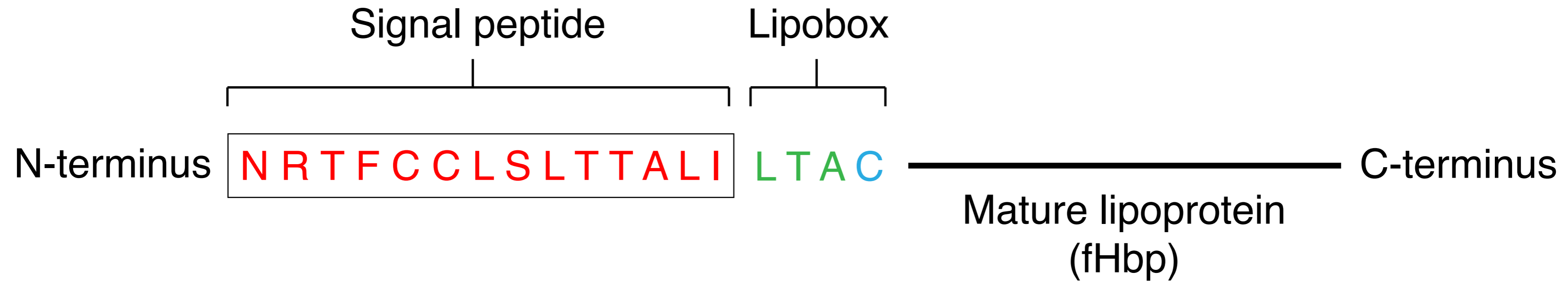

Supplement: Supplementary file 13 — Additional file 13: Figure S1. Schematic of the N-terminal full-length signal sequence upstream of the factor H binding protein lipoprotein sequence. [file 13578_2022_809_MOESM13_ESM.pdf]

**A**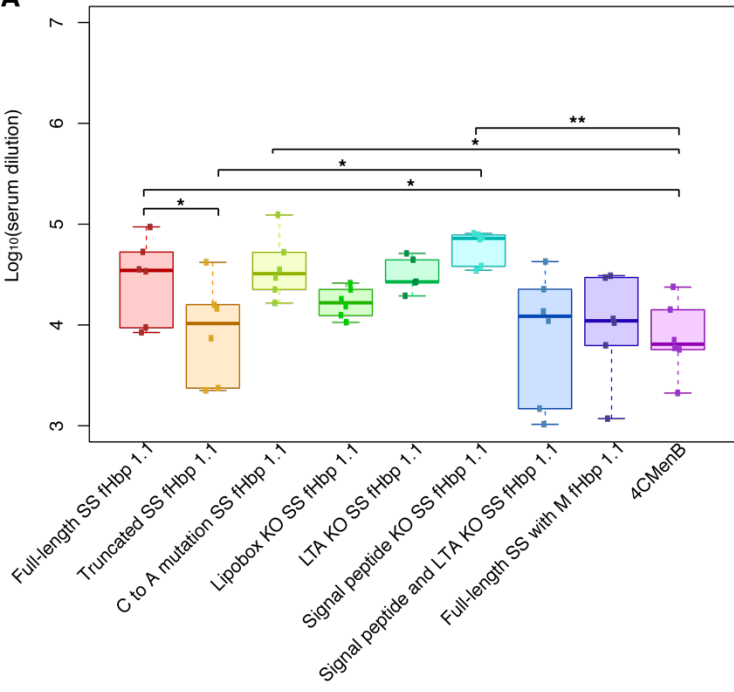

Supplement: Supplementary file 14 — Additional file 14: Figure S2. Antigen-specific IgG titres in sera from mice immunised with human adenovirus vectors encoding N-terminal sequence variants of the factor H binding protein antigen. Groups of six BALB/c mice were immunised with a sub-optimal dose of 1 × 10^7^ infectious units of one of the human adenovirus serotype 5 (AdHu5) vectors encoding factor H binding protein (fHbp) with N-terminal signal sequence variants or 1/10 of the human dose of 4CmenB as a comparator. Enzyme-linked immunosorbent assays were performed on serum samples taken at weeks (A) two, (B) four, (C) six, and (D) 14 post-immunisation to determine the titres of anti-fHbp IgG in sera. Statistical comparisons were made using a Mann-Whitney U-test. * p < 0.05; ** p < 0.01; *** p < 0.001. [file 13578_2022_809_MOESM14_ESM.pdf]

## Surface vs. intracellular staining

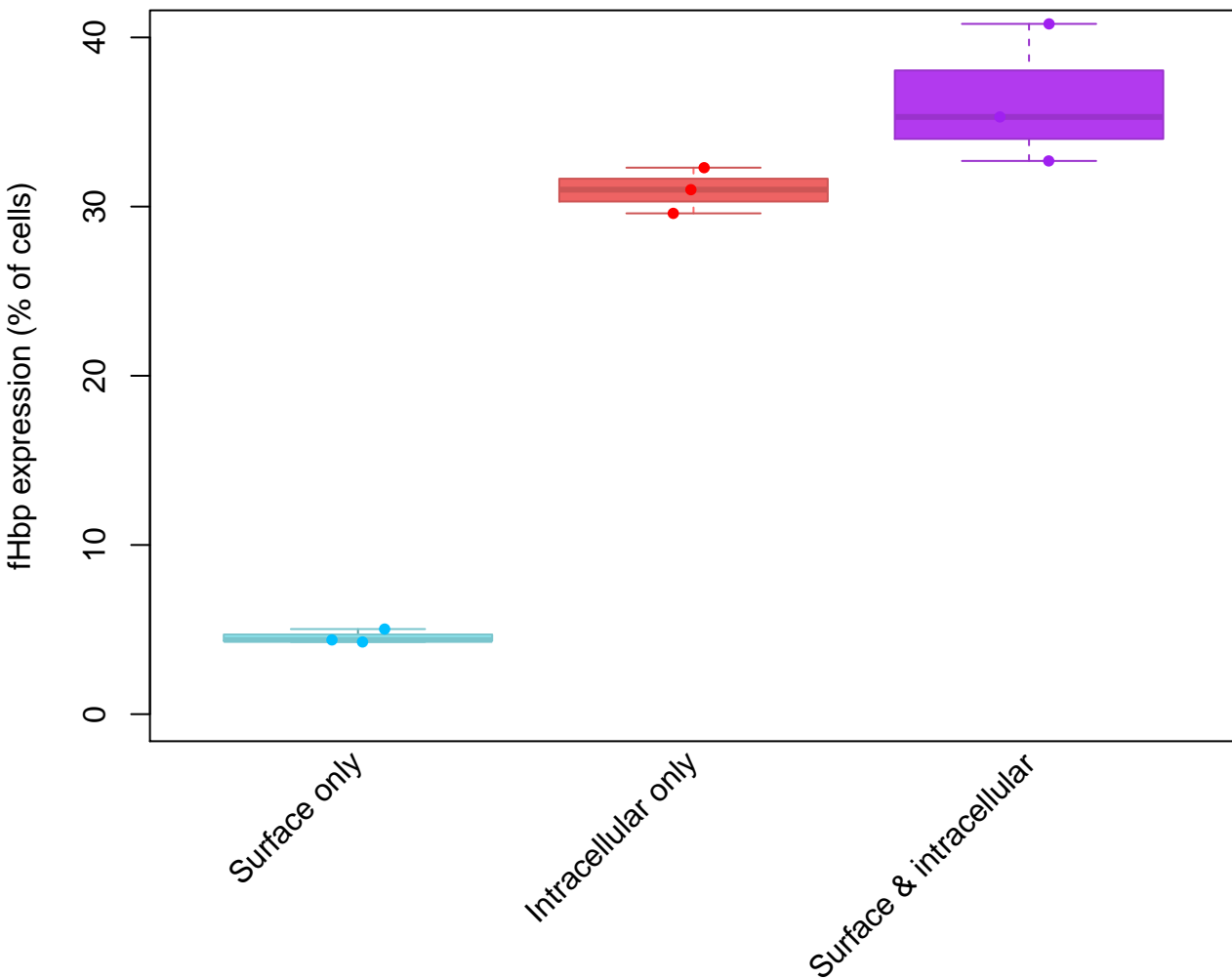

Supplement: Supplementary file 15 — Additional file 15: Figure S3. Intracellular and surface expression of human adenovirus-encoded factor H binding protein with a full-length N terminal signal sequence on HeLa cells after overnight infection. HeLa cells (1 × 10^6^ per sample) were infected overnight with 5 × 10^8^ infectious units of a human adenovirus serotype 5 (AdHu5) construct encoding the factor H binding protein (fHbp) with a full-length signal sequence and expression was quantified by flow cytometry after surface only, intracellular only, or both surface and intracellular staining of harvested cells with an anti-fHbp antibody (JAR5) and a fluorescently-tagged detection antibody. The y-axis corresponds to the percentage of total fluorescent (fHbp-expressing) HeLa cells after overnight infection. The vast majority of antigen is expressed within the cell at this timepoint. [file 13578_2022_809_MOESM15_ESM.pdf]

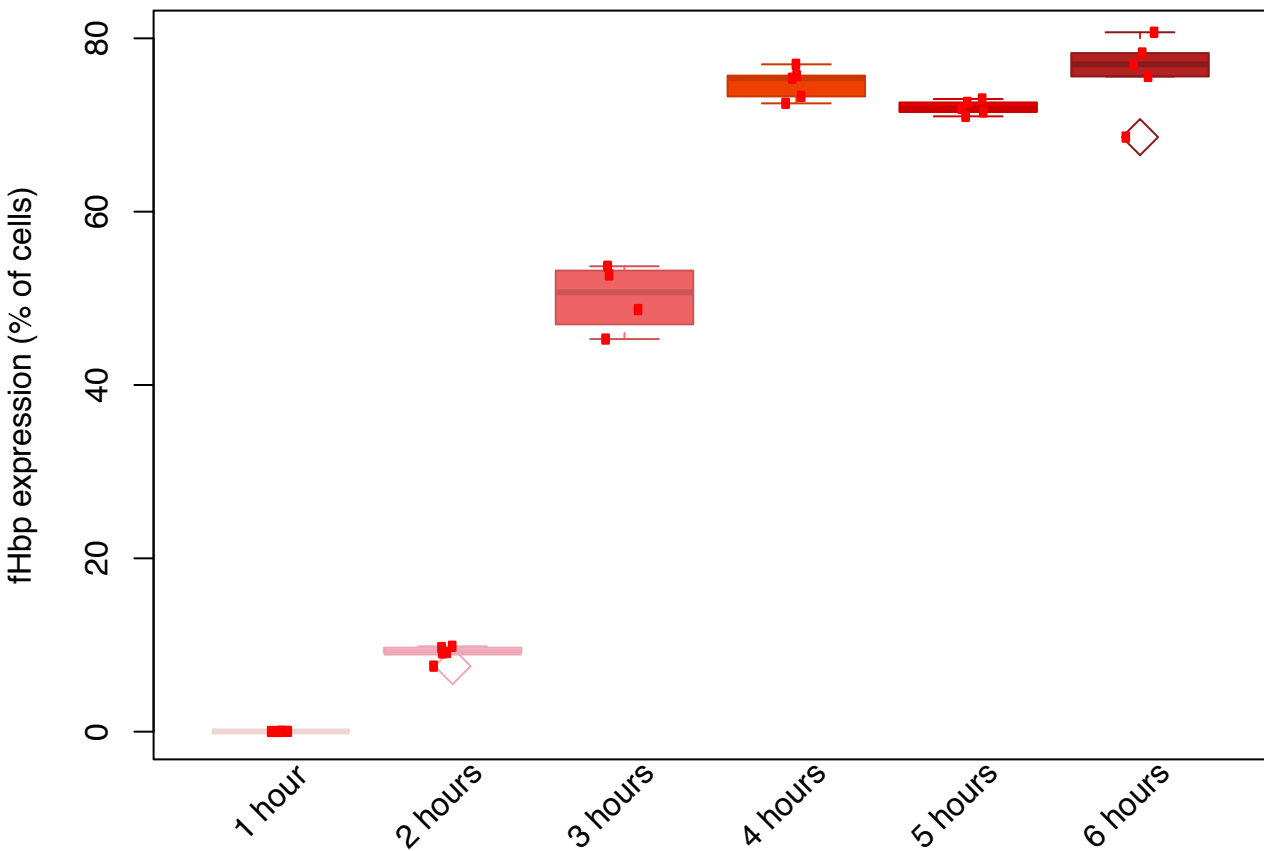

Supplement: Supplementary file 16 — Additional file16: Figure S4. Time-course expression assay of human-adenovirus-encoded factor H binding protein with a full-length N-terminal signal sequence on HeLa cells stimulated with brefeldin. A. HeLa cells (1 × 10^6^ per sample) were stimulated with brefeldin A to stop protein transport within the cells and subsequently infected with 5 × 10^8^ infectious units of a human adenovirus serotype 5 (AdHu5) construct encoding the factor H binding protein (fHbp) with a full-length signal sequence and expression was quantified by flow cytometry after intracellular only staining of harvested cells with an anti-fHbp antibody (JAR5) and a fluorescently-tagged detection antibody. The y-axis corresponds to the percentage of total fluorescent (fHbp-expressing) HeLa cells after each hourly timepoint post-infection (x-axis) up to six hours. The antigen begins to be expressed at high levels at three hours post-infection and expression levels plateau around five hours. [file 13578_2022_809_MOESM16_ESM.pdf]

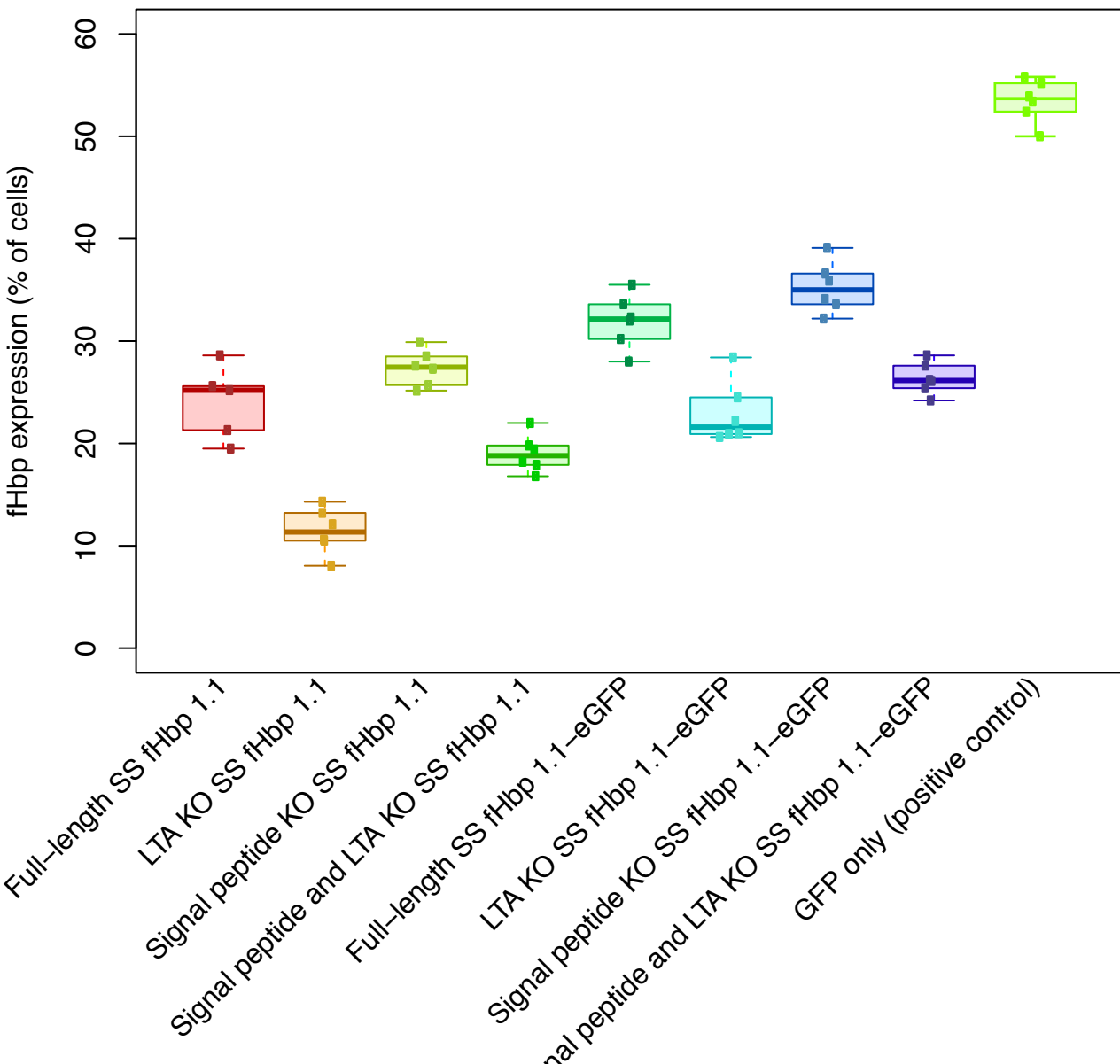

Supplement: Supplementary file 17 — Additional file17: Figure S5. Side-by-side comparison of factor H binding protein N-terminal signal sequence variants, with and without enhanced green fluorescent protein tags, expressed from human adenovirus vectors after overnight infection HeLa cells. HeLa cells (1 × 10^6^ per sample) were infected overnight with 5 × 10^8^ infectious units of one of a series of human adenovirus serotype 5 (AdHu5) constructs encoding an N-terminal sequence variant of the factor H binding protein (fHbp), fHbp fused with enhanced green fluorescent protein (eGFP), or GFP only, and expression was quantified by flow cytometry after surface and intracellular staining of non-GFP-expressing cells with an anti-fHbp antibody (JAR5) and a fluorescently-tagged detection antibody. The y-axis corresponds to the percentage of total fluorescent (fHbp- and/or GFP-expressing) HeLa cells after overnight infection. The differences in expression levels between the antigen variants tested is conserved for the eGFP fusion antigens and lower for the eGFP fusion antigens than the eGFP only positive control, confirming that the differences in antigen expression levels are attributable to the fHbp N-terminal signal sequence. [file 13578_2022_809_MOESM17_ESM.pdf]
